# Fatal Wunderlich Syndrome During Hemodialysis: A Rare Case and Review of the Literature

**DOI:** 10.7759/cureus.110573

**Published:** 2026-06-10

**Authors:** Darius Mbina Nzienghi, Liela Agnès Okoyi Ossouka, Yahya Sqalli Houssaini, Said Jidane, Tahir Nebhani, Ahmed Belkouch, Lahcen Belyamani

**Affiliations:** 1 Department of Emergency Medicine, Mohammed V Military Training Hospital, Rabat, MAR; 2 Department of Emergency Medicine, Mohammed V University, Rabat, MAR; 3 Department of Radiology, Mohammed V Military Training Hospital, Rabat, MAR

**Keywords:** critical emergency medicine, hemodialysis, lenk’s triad, spontaneous renal hemorrhage, wunderlich syndrome

## Abstract

Wunderlich syndrome (WS) is a rare but life-threatening condition characterized by spontaneous, non-traumatic renal hemorrhage, which may present with Lenk’s triad. Early recognition and prompt management are crucial to improve prognosis. We report the case of a 49-year-old man with end-stage renal disease secondary to antineutrophil cytoplasmic antibody-associated vasculitis on maintenance hemodialysis who developed sudden left flank pain during a routine hemodialysis session, without preceding trauma. Laboratory evaluation revealed anemia and elevated international normalized ratio. Computed tomography demonstrated an enlarged left kidney with multiple cystic lesions and a large perirenal hematoma extending into the retroperitoneal space, consistent with WS. Despite initial resuscitative measures, the patient rapidly deteriorated into refractory hemorrhagic shock and died before definitive intervention could be performed. This case highlights the potential for rapid deterioration in hemodialysis patients and underscores the importance of early recognition, rapid imaging, and timely multidisciplinary management.

## Introduction

Wunderlich syndrome (WS), or spontaneous renal hemorrhage, is defined as non-traumatic retroperitoneal bleeding of renal origin. Although uncommon, it carries high mortality if diagnosis is delayed, with reported rates up to 20-30% [1]. Its presentation includes Lenk’s triad of acute flank or abdominal pain, palpable mass, and hypotension, although the complete triad is present in only about 20% of patients; more often, symptoms are nonspecific, including nausea, vomiting, fever, or hematuria [1]. The main etiologies include renal angiomyolipoma (60-66%) and renal cell carcinoma (30-35%). Less common causes include coagulopathies, vascular malformations, and acquired cystic kidney disease (ACKD) [2,3]. Patients undergoing chronic hemodialysis represent a high-risk group due to uremic vasculopathy, fragile cystic vessels in ACKD, and the use of anticoagulation during dialysis. The reported incidence of WS among patients receiving hemodialysis is less than 1%, with only a limited number of case reports and small series addressing this population [4]. However, data specifically addressing the occurrence of WS during hemodialysis sessions remain extremely limited. We describe a fatal case characterized by rapid hemodynamic deterioration, emphasizing the narrow therapeutic window in such patients.

## Case presentation

A 49-year-old male presented to our emergency department one hour after the onset of sudden and severe left flank pain that occurred during the third hour of his routine hemodialysis session. He denied any history of recent trauma, fever, dysuria, or other urinary symptoms. The patient had a history of end-stage renal disease (ESRD) secondary to antineutrophil cytoplasmic antibody (ANCA)-associated vasculitis and had been receiving thrice-weekly hemodialysis for the past three years. During hemodialysis sessions, he routinely received standard intradialytic anticoagulation with unfractionated heparin to prevent coagulation of the extracorporeal circuit and dialyzer rather than for systemic anticoagulation. No history of anticoagulant overdose or liver disease was identified.

On arrival, the patient was pale, diaphoretic, and in significant distress due to pain, but remained fully conscious and oriented. Initial vital signs showed a blood pressure (BP) of 102/64 mmHg, a heart rate (HR) of 98 beats/minute, and an oxygen saturation of 97% on room air. Physical examination revealed left flank tenderness without guarding or palpable mass. There were no ecchymoses or signs of external trauma.

Laboratory investigations demonstrated normocytic anemia (hemoglobin: 8.6 g/dL; normal range (NR): 12.0-16.0 g/dL), leukocytosis (14,000/μL; NR: 4,000-11,000/μL), and a markedly elevated international normalized ratio (INR) of 4.26 (NR: 0.8-1.2). Renal function tests showed an elevated blood urea of 57 mg/dL (NR: 15-45 mg/dL) and a serum creatinine of 5.1 mg/dL (NR: 0.6-1.2 mg/dL). Platelet count was within the normal range at 181,000/μL (NR: 150,000-450,000/μL), and liver biochemistry was within normal limits. C-reactive protein was also within normal limits at 4.8 mg/L (NR: <5.0 mg/L).

Bedside point-of-care ultrasound (POCUS) demonstrated a hyperechoic perirenal collection surrounding the left kidney, consistent with a perirenal hematoma, with no intraperitoneal fluid.

Given the suspicion of WS, immediate resuscitative measures were initiated, including administration of a 250 mL crystalloid bolus, transfusion of one unit of packed red blood cells, intravenous calcium gluconate (1 g), and prothrombin complex concentrate at a dose of 50 IU/kg.

Non-contrast computed tomography (CT) demonstrated an enlarged left kidney with multiple cystic lesions and a hyperdense perirenal hematoma extending into the retroperitoneal space, highly suggestive of WS (Figures 1, 2).

**Figure 1:**
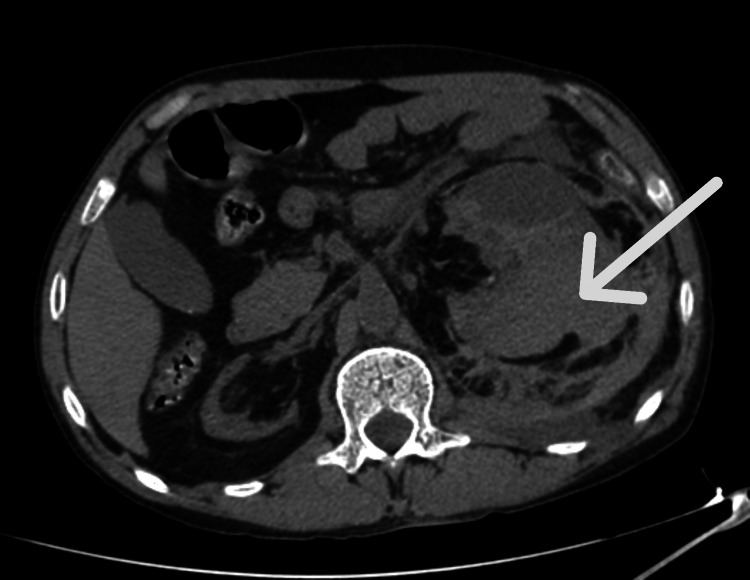
Axial non-contrast abdominal computed tomography scan showing a hyperdense left renal and perirenal hematoma (white arrow), with extension into the perirenal space and surrounding retroperitoneal fat.

**Figure 2:**
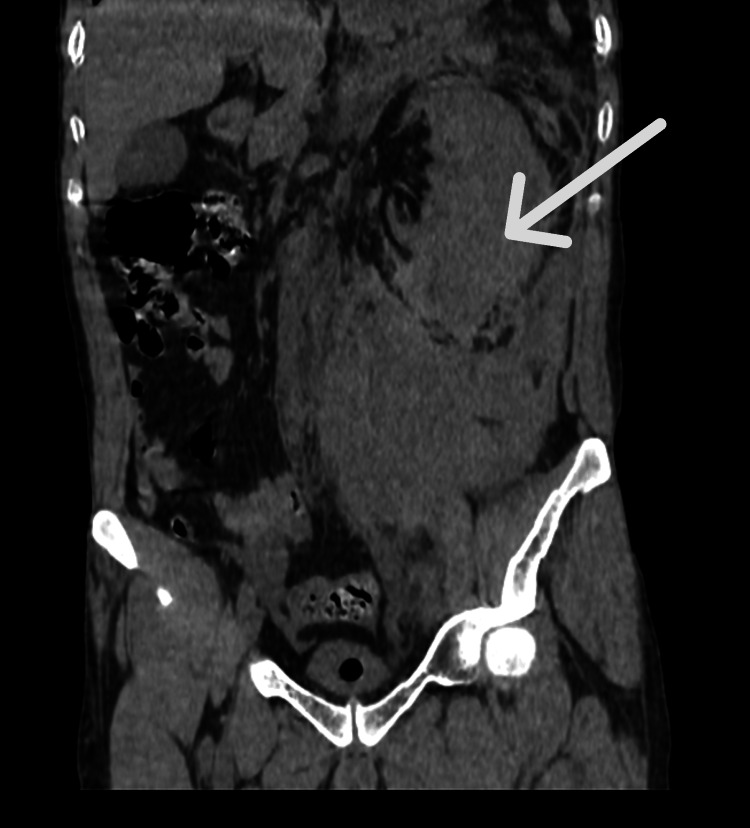
Coronal reconstruction of a non-contrast abdominal computed tomography scan showing a left renal and perirenal hematoma (white arrow) with associated retroperitoneal extension.

Following CT imaging, the patient’s condition rapidly deteriorated into hemorrhagic shock, prompting escalation of resuscitative efforts with a strategy of permissive hypotension while preserving adequate organ perfusion. Norepinephrine infusion (0.05-0.1 µg/kg/minute, titrated according to mean arterial pressure) was initiated, and additional blood products, including packed red blood cells, fresh frozen plasma, and platelets, were urgently requested to support ongoing resuscitation. Interventional radiology and surgical teams were immediately consulted.

Despite escalation of resuscitative efforts, the patient remained hemodynamically unstable. Given the refractory shock and the anticipated delay to endovascular embolization, emergent surgical exploration was deemed the most appropriate definitive management strategy. Before transfer to the operating room could be completed, the patient suffered a cardiac arrest approximately 50 minutes after emergency department admission. Advanced cardiopulmonary resuscitation was initiated but was unsuccessful, and death was pronounced after 30 minutes of resuscitative efforts.

The patient’s clinical course, interventions, and outcomes are summarized in Table 1, providing a chronological timeline from symptom onset during hemodialysis to death.

**Table 1:** Timeline of the patient’s clinical events. ED = emergency department; CPR = cardiopulmonary resuscitation

Time point	Clinical event
Hour 3 of hemodialysis	Sudden severe left flank pain during hemodialysis; Hemodialysis session interrupted; patient transferred to our emergency department.
+1 hour	Arrival at our emergency department; pale, BP 102/64 mmHg, HR 98/min.
During ED stay	POCUS suggestive of a perirenal hematoma; CT confirmed Wunderlich syndrome; progressive hypotension and development of hemorrhagic shock.
+50 minutes after ED admission	Cardiac arrest.
Next 30 minutes	CPR performed for 30 minutes.
~1 hour 20 minutes after ED admission	Death declared.

## Discussion

First described by Carl Reinhold August Wunderlich in 1856, WS refers to spontaneous, non-traumatic renal hemorrhage into the subcapsular and/or perirenal space and remains a rare but life-threatening emergency [1]. Its clinical presentation is often heterogeneous, but it should be strongly suspected in patients presenting with sudden flank or abdominal pain associated with hypotension, particularly in high-risk populations such as those undergoing chronic hemodialysis [2-4].

As emphasized by Shah et al., contrast-enhanced CT remains the gold standard for the diagnosis of WS, as it allows accurate identification of active contrast extravasation and underlying renal pathology [1]. In our case, an initial non-contrast CT scan was performed immediately after POCUS to rapidly confirm the presence and extent of retroperitoneal hemorrhage. Although contrast-enhanced CT would have provided additional information regarding active bleeding and possible underlying etiologies, the non-contrast study was sufficient to establish the diagnosis and guide immediate management. Given the patient’s subsequent rapid hemodynamic deterioration, additional contrast-enhanced imaging could not be performed. Therefore, the absence of contrast represented a limitation for etiological assessment rather than for initial diagnostic confirmation.

The etiology of WS is diverse, with renal neoplasms, particularly angiomyolipoma and renal cell carcinoma, representing the most frequently reported causes [1,2]. In their retrospective analysis of 46 cases of spontaneous perirenal hemorrhage, Jo et al. similarly identified renal tumors as the predominant etiology, followed by ACKD and idiopathic causes [3]. Other recognized contributors include coagulopathies, anticoagulation therapy, and vascular fragility, especially in patients with chronic kidney disease [2,3]. Hemodialysis patients represent a particularly vulnerable subgroup because of the convergence of several predisposing factors, including uremic vasculopathy, repeated anticoagulation exposure, recurrent hemodynamic fluctuations, and the high prevalence of ACKD [4,5]. Malek-Marín et al. notably highlighted ACKD and anticoagulation as major contributors to spontaneous retroperitoneal hemorrhage in dialysis patients [4]. In our case, the patient exhibited several predisposing factors, including underlying vasculopathy and renal cysts consistent with ACKD, further compounded by hemodynamic fluctuations and anticoagulation exposure inherent to hemodialysis sessions.

A markedly elevated INR of 4.26 was observed on admission in the absence of vitamin K antagonist therapy. Liver biochemistry was within normal limits, and there was no clinical or laboratory evidence of disseminated intravascular coagulation or thrombocytopenia, making primary hepatic failure and overt disseminated intravascular coagulation unlikely. In this context, the coagulopathy was likely multifactorial, reflecting critical illness-associated coagulopathy and intradialytic anticoagulation exposure. Although uremia primarily causes platelet dysfunction rather than prothrombin time/INR prolongation, it may have further increased the bleeding tendency. Exposure to unfractionated heparin during dialysis may also have contributed to the bleeding risk; however, heparin alone would not be expected to account for such a marked elevation in INR. The exact dose and timing of unfractionated heparin administered during the dialysis session were unavailable, which represents a limitation. Consequently, the potential role of protamine reversal could not be adequately assessed. Although vitamin K deficiency could not be definitively confirmed, it remains a plausible contributing factor in patients with end-stage renal disease and impaired nutritional status. Taken together, these factors likely contributed to the profound hemorrhagic susceptibility observed in the present case.

Management of WS is primarily guided by the patient’s hemodynamic status and the underlying etiology, ranging from conservative treatment with transfusion and close monitoring in stable patients to selective arterial embolization and surgical management, including nephrectomy, in refractory or life-threatening cases [1,6]. Recent literature increasingly supports embolization as the preferred first-line intervention in hemodynamically stable or stabilized patients because of its minimally invasive nature and effectiveness in controlling active bleeding [6,7]. Early recognition and prompt intervention remain critical determinants of prognosis [2]. In our case, symptoms developed during a routine hemodialysis session at an outpatient dialysis center. Dialysis was immediately discontinued, and the patient was promptly transferred to our emergency department. Because the dialysis center was located in a rural area, the patient arrived approximately one hour after symptom onset. No specific treatment was administered before transfer. Despite an initially non-critical presentation, the patient’s condition worsened rapidly, underscoring the fulminant nature of WS and the narrow therapeutic window for definitive management.

While WS is an uncommon clinical entity, its occurrence during hemodialysis is even rarer, with very few cases reported during dialysis sessions, particularly those with fatal outcomes. To better contextualize our findings, a focused literature review was performed using PubMed/MEDLINE and Google Scholar. Databases were searched up to April 2026 using combinations of the terms “Wunderlich syndrome,” “spontaneous renal hemorrhage,” and “hemodialysis.” Articles published in English or French were considered. Only reports describing WS occurring during hemodialysis were included. References from relevant articles were also screened to identify additional eligible cases, whereas reports lacking sufficient clinical information or involving non-hemodialysis patients were excluded. The previously reported cases identified through this search are summarized in Table 2 [6-10].

**Table 2:** Case reports of Wunderlich syndrome occurring during hemodialysis. *: Delayed death from an unrelated complication. N/A = not available; ACKD = acquired cystic kidney disease; ED = emergency department; ESRD = end-stage renal disease; HD = hemodialysis

Author	Age (years)	Sex	Clinical presentation	Onset to ED time	Predisposing factors	Management	Outcome
Abidi et al. [6]	26	F	Acute right flank pain	N/A	ESRD on HD; anticoagulation	Embolization right nephrectomy	Discharged
Xie et al. (case number 2) [7]	45	F	Acute left abdominal pain	N/A	ESRD on HD; anticoagulation	Embolization	Discharged
Murphy et al. [8]	55	M	Acute left abdominal pain, hypotension	N/A	ESRD on HD; ACKD	Left nephrectomy	Discharged
Roxo et al. [9]	56	M	Acute left flank pain, hypotension	N/A	ESRD on HD; anticoagulation; ACKD	Left nephrectomy	Discharged
Bluhm et al. [10]	79	M	Acute left abdominal pain	30 minutes	ESRD on HD; renal cyst	Embolization	Discharged*
Our case	49	M	Acute left flank pain	1 hour	ESRD on HD; anticoagulation; ACKD	Initial resuscitation	Death

Most reported patients had favorable outcomes following embolization or surgical intervention. In contrast, our case illustrates that WS occurring during hemodialysis may, in certain circumstances, follow a particularly aggressive course characterized by rapid hemodynamic deterioration and a very narrow therapeutic window. This course may be further exacerbated by hemodynamic fluctuations and anticoagulation exposure inherent to dialysis sessions. The fatal outcome observed in our patient underscores the importance of early diagnosis and highlights the potential consequences of delayed management. Early recognition at the dialysis unit level, followed by prompt transfer to a facility capable of definitive hemorrhage control, may be critical to improving outcomes.

## Conclusions

WS occurring during hemodialysis is an exceptionally rare but potentially catastrophic condition characterized by rapid clinical deterioration and a narrow therapeutic window. Sudden flank pain occurring during hemodialysis should be considered a potential sentinel symptom of retroperitoneal hemorrhage. In such cases, prompt interruption of dialysis with immediate discontinuation of anticoagulation, urgent cross-sectional imaging, and early multidisciplinary involvement with interventional radiology and surgical teams may help improve outcomes. Increased awareness among dialysis staff and emergency physicians may facilitate earlier diagnosis and timely definitive management in this high-risk population.
